# Supplementary material for: MetaRibo-Seq measures translation in microbiomes
Source: Nat Commun. 2020 Jun 29;11:3268. doi: 10.1038/s41467-020-17081-z (PMC7324362; doi:10.1038/s41467-020-17081-z)
Supplement: Supplementary file 10 — Supplementary Data 7 [file 41467_2020_17081_MOESM10_ESM.zip › File2/Confidence_High_Taxonomy/12841_out.krona.html]

Javascript must be enabled to view this page.

members
magnitude
magnitudeUnassigned
count
unassigned
taxon
rank

12841\_out

3

2
3
superkingdom

1239
3
phylum

class
3
186801

order
186802
3

SRS043411\_contig\_number\_contig-100\_10344.10344SRS058770\_contig\_number\_contig-100\_32549.72566SRS063040\_contig\_number\_contig-100\_5078.270068
